# The triglyceride–glucose index is associated with no-reflow phenomenon in STEMI patients with type 2 diabetes after percutaneous coronary intervention

**DOI:** 10.3389/fcvm.2024.1386318

**Published:** 2024-09-12

**Authors:** Juan Ma, Peng Wu, Shengzong Ma, Xueping Ma, Ping Jin, Shaobin Jia

**Affiliations:** ^1^ School of Clinical Medicine, Ningxia Medical University, Yinchuan, China; ^2^ Department of Gynaecology and Obstetrics, People’s Hospital of Ningxia Hui Autonomous Region, Yinchuan, China; ^3^ Heart Centre & Department of Cardiovascular Diseases, General Hospital of Ningxia Medical University, Yinchuan, China; ^4^ Department of Cardiology, Second Affiliated Hospital of Xi’an Jiaotong University, Xi’an, Shaanxi, China; ^5^ Institute of Medical Sciences, General Hospital of Ningxia Medical University, Yinchuan, China

**Keywords:** insulin resistance (IR), triglyceride–glucose index (TyG index), type 2 diabetes mellitus (T2DM), ST-segment elevation myocardial infarction (STEMI), no-reflow phenomenon (NRP)

## Abstract

**Background:**

The relationship between the triglyceride–glucose (TyG) index and no-reflow phenomenon after percutaneous coronary intervention (PCI) in patients with type 2 diabetes mellitus (T2DM) and acute ST-segment elevation myocardial infarction (STEMI) remains unclear. This study aimed to investigate the relationship between baseline TyG index and no-reflow phenomenon in STEMI patients with T2DM after PCI.

**Methods:**

This study enrolled 695 patients with T2DM and STEMI from the General Hospital of Ningxia Medical University (2014–2019). Patients were divided into tertiles according to the TyG index levels. The incidence of no-reflow phenomenon was recorded. A multivariate regression model was developed to analyze the association between the baseline TyG index and no-reflow phenomenon. The linear association between the baseline TyG index and no-reflow phenomenon was explored using smooth curve fitting with parallel subgroup analyses. Receiver operating characteristic (ROC) curves were generated to determine the predictive power of the TyG index.

**Results:**

A multivariate logistic regression model revealed that the TyG index was an independent risk factor of no-reflow phenomenon [OR = 3.23, 95%CI: 2.15–4.86, *P* < 0.001], and the occurrence of no-reflow phenomenon increased gradually with the increase of TyG index tertile interval (*P* < 0.001). Smooth curve fitting showed that the TyG index was linearly related to the risk of no-reflow. Subgroup analysis showed that they participated in this positive correlation. The area under the ROC curve (AUC) of the TyG index for evaluating the occurrence of no-reflow was 0.710 (95% CI: 0.640–0.780; *P < *0.01).

**Conclusions:**

The TyG index is independently associated with no-reflow phenomenon, suggesting that the simple index of the TyG index can be used for risk assessment of no-reflow phenomenon after PCI in STEMI patients with T2DM.

## Background

Myocardial perfusion should be re-established as soon as possible in patients with acute ST-elevation myocardial infarction (STEMI) (1). Primary percutaneous coronary intervention (PCI) is the modality of choice for restoring myocardial blood flow, which significantly prevents further necrosis of cardiomyocytes and markedly improves the quality of life of STEMI patients (2). However, even after the culprit vessel is reopened, there may be suboptimal coronary reperfusion, with slow, incomplete, or no coronary flow in the affected coronary arteries based on a thrombolysis in myocardial infarction (TIMI) score of <3 (3). This phenomenon, known as “no-reflow” (NR), occurs in 2%–60% of patients and can complicate up to 60% of STEMI cases (4). No-reflow is associated with an increased incidence of rehospitalization, adverse ventricular remodeling, malignant arrhythmias, and heart failure and is an independent predictor of cardiovascular mortality (5). This risk is particularly high in patients with type 2 diabetes mellitus (T2DM), which accounts for approximately 37% of acute myocardial infarction (AMI) cases in China and is considered a high-risk group for recurrent cardiovascular mortality (6). A previous study has shown that T2DM is significantly associated with more complex coronary artery lesions and a higher incidence of no-reflow in STEMI patients (7). Therefore, early identification of residual risk factors in STEMI patients with T2DM is essential for better clinical management to reduce the incidence of future no-reflow.

Insulin resistance (IR) is a state of reduced sensitivity and responsiveness of the body to insulin, usually occurring in the prediabetic and diabetic stages (8). The hyperinsulinemic–euglycemic clamp is the gold standard test for assessing IR, but it is not widely used in clinical care and large population studies due to the complexity and cost of the test procedure (9). The Homeostatic Model Assessment of Insulin Resistance (HOMA-IR) is a method used to quantify IR and beta-cell function, which is calculated using fasting glucose and insulin levels, providing an estimate of IR that is crucial in understanding the metabolic profile of patients. The triglyceride–glucose (TyG) index, a composite index calculated as Ln fasting triglycerides (mg/dl) × fasting plasma glucose (FPG) (mg/dl)/2, is not only inexpensive and readily available but also performs consistently with or better than HOMA-IR in the assessment of IR (10). In recent studies, the TyG index has been widely used as a marker of IR. It has been shown that a high TyG index was associated with an increased risk of major adverse cardiac and cerebrovascular events in STEMI patients undergoing PCI (11) and the risk of ischemic stroke was associated with a proportional and linear increase in TyG index (12). However, no previous study has specifically examined the association of the TyG index with the no-reflow after PCI in STEMI patients with T2DM. Our study was designed to fill this knowledge gap.

## Methods

### Study population

**Figure 1:**
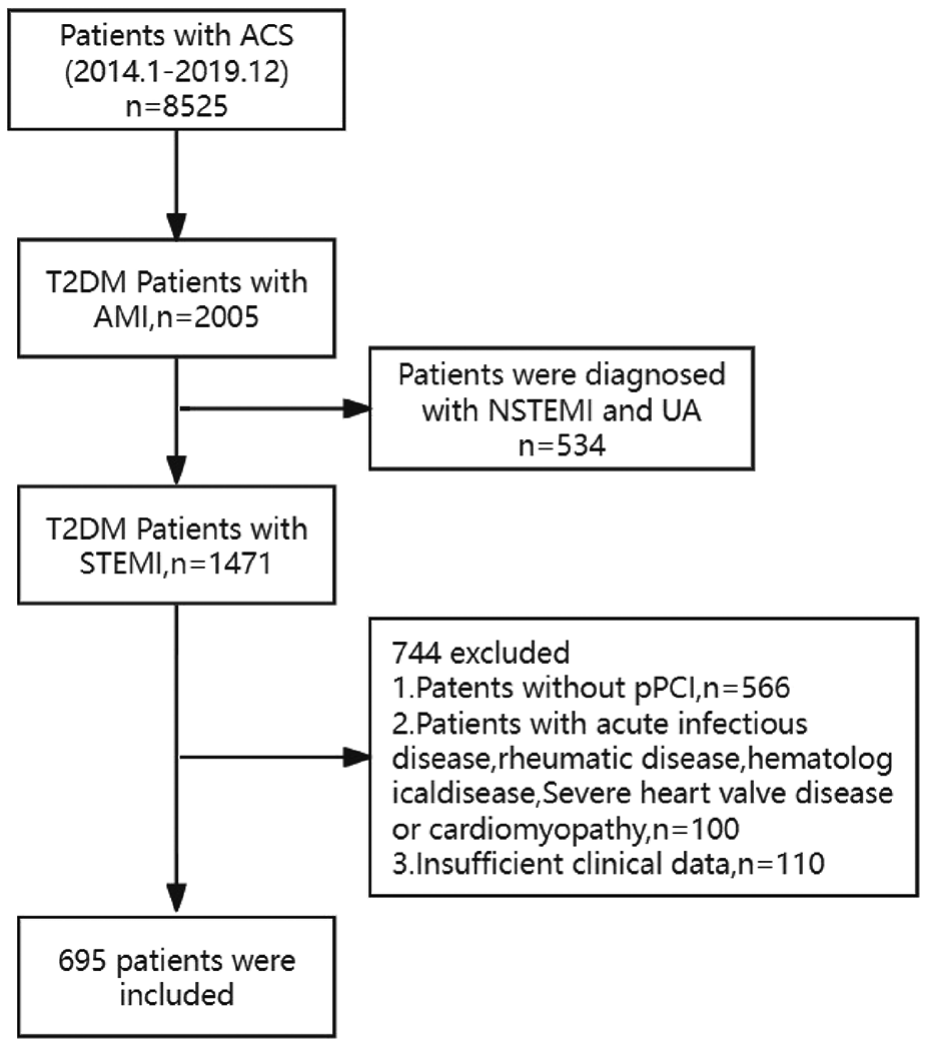
The flowchart of the research subject. ACS, acute coronary syndrome; AMI, acute myocardial infarction; STEMI, acute ST-elevation myocardial infarction; PCI, primary percutaneous coronary intervention; T2DM, type 2 diabetes mellitus.

The study subjects were screened from the database of the cardiovascular center of the General Hospital of Ningxia Medical University. The patient flowchart is presented in Figure 1. A total of 8,525 consecutive patients were diagnosed with acute coronary syndrome (ACS) from January 2014 to December 2019. Of these 8,525 patients, 1,471 were diagnosed with STEMI and T2DM. A total of 776 patients were excluded according to the exclusion criteria, including (1) without primary PCI; (2) with acute infectious disease, rheumatic disease, or hematological disease; (3) with severe heart valve diseases or cardiomyopathy; and (4) lacking clinical data. Finally, 695 patients were included in this analysis. According to the TyG index level, 695 patients were stratified into four groups (Q1 group: 6.25 < TyG index <8.15, *n* = 174; Q2 group: 8.15 ≤ TyG index <8.68, *n* = 173; Q3 group:8.68 ≤ TyG index <9.29, *n* = 174; Q4 group:9.29 ≤ TyG index <11.34, *n* = 174).

### PCI procedure and angiographic analysis

We used the standard PCI procedure: a thorough preoperative evaluation was carried out, with an episodic dose of 300 mg clopidogrel, 180 mg ticagrelor, and 300 mg aspirin and total heparinization. Selective coronary angiography and infarct-related arteriography (IRA) were performed after puncture of the right radial artery using the conventional Seldinger technique. The optimal interventional transport device was selected to deliver the stent and the angiogram was reviewed. Success criteria for PCI treatment: TIMI grade 3 under direct vision. The decision to administer intracoronary adenosine, intravenous tirofiban, and other drugs is made by the interventionalist based on the patient's specific coronary artery lesions.

### Data collections and definitions

The data collection process was approved by the Institutional Review Board of the General Hospital of Ningxia Medical University and was in accordance with the Regulations on the Review of Medical Institutions.

Patient demographics, medical history, laboratory findings, and echocardiographic and angiographic assessments were collected and validated from our hospital's electronic medical record system. The concentrations of triglyceride (TG) and fasting plasma glucose (FPG) in the first fasting blood sample during hospitalization were determined in the central laboratory of the General Hospital of Ningxia Medical University. The TyG index was calculated as ln[fasting TG (mg/dl) × FPG (mg/dl)/2] (13). The single-point insulin sensitivity estimator (SPISE) has been proven to be an effective surrogate index for insulin sensitivity, so it is included in the baseline clinical characteristics. The novel formula for SPISE was computed as follows: SPISE= $600\;\times \;\mathrm{HDL}-C^{0.185}/\left[TG^{0.2}\;\times \;\mathrm{body}\;\mathrm{mass}\;\mathrm{index}{\left(\mathrm{BMI}\right)}^{1.338}\right]$ .

Definition of the no-reflow phenomenon: For the purpose of this study, the diagnosis of the no-reflow phenomenon was by angiography during the PCI procedure by two independently experienced interventionalists. We defined no-reflow as a TIMI grade flow of ≤2 after coronary reopening.

Criteria for T2DM included: (1) a previous diagnosis of T2DM and being treated with antidiabetic medication and (2) FPG ≥7.0 mmol/L and/or random blood glucose (RBG) ≥11.1 mmol/L and/or blood glucose ≥11.1 mmol/L 2 h after an oral glucose tolerance test (OGTT) as a typical symptom of DM.

### Statistical analyses

Empower software (http://www.empowerstats.com) and R version 4.1.0 (http://www.R-project.org) were employed for all analyses. Study participants were categorized into four groups based on quartiles (Q1–Q4) of the TyG index. Non-parametric Kruskal–Wallis ANOVA was used to compare the sample means of the four groups in fully randomized experimental design data when the study data population was not normally distributed and/or the variance was not homogeneous. For continuous variable data in the randomized area group design, the Friedman rank sum test was used when the experimental groups were not normally distributed. Once a significant difference was found, a *post hoc* test was performed to determine which specific treatment groups differed from each other, using the Dunnett validated randomization test. A multivariate logistic regression model was used to analyze the correlation between the TyG index and no-reflow. The correlation between the TyG index and no-reflow was further evaluated by smooth curve fitting. Stratified analyses were conducted based on gender, age (<65 or ≥65 years), hypertension, dyslipidemia, current smoker, BMI (<25.00 or ≥25.00), and eGFR (<60 or ≥60 ml/min/1.73 m^2^). Receiver operating characteristic (ROC) curves were generated to determine the cutoff values and predictive power of the TyG index, HbA1c, and the SPISE index. All statistical tests were two-tailed, and a significance level of *P* < 0.05 was considered statistically significant.

## Results

### Baseline characteristics of study participants

Table 1 shows the baseline characteristics of the patients (*n* = 695) stratified by TyG index quartiles. The average age of the patients was 61.57 years, and 71.08% of them were male. The average TyG index in the enrolled patients was 8.71 ± 0.83. Compared with the lowest quartile, the participants with a higher TyG index were more likely to be younger and showed a higher body mass index (BMI) and higher percentage of previous dyslipidemia (all *P *< 0.05). Additionally, the laboratory and angiographic characteristics at baseline are shown in Table 2 according to the TyG index quartiles. The participants in the highest quartile showed significantly higher levels of TyG index, hemoglobin A1c (HbA1c), total cholesterol(TC), TG, FPG, and albumin compared with those of the participants in the first quartile. However, the SPISE index and HDL cholesterol and hemoglobin levels of the participants in the highest quartile were lower than those of the participants in the first quartile (all *P* < 0.05). Angiographically, the participants in the highest quartile had a significantly higher percentage of no-reflow than that of the participants in the lowest quartile (*P < *0.001).

**Table 1:** Baseline characteristics according to the TyG index quartiles.

Characteristics	Quartiles of the TyG index					*F*-value	*P*-value
	Overall	Q1 (6.25–8.15)	Q2 (8.15–8.68)	Q3 (8.68–9.29)	Q4 (9.29–11.34)		
N	695	174 (25.04%)	173 (24.89%)	174 (25.04%)	174 (25.04%)		
Age, years	61.57 (10.61)	64.25 (9.45)	62.65 (10.24)	60.76 (10.84) ^b^	58.63 (11.04)^c,e^	9.454	<0.001
Male	494 (71.08%)	127 (72.99%)	125 (72.25%)	117 (67.24%)	125 (71.84%)	0.572	0.632
BMI, kg/m^2^	25.13 (3.55)	24.6 (2.8)	24.9 (4.0)	25.1 (3.3)	26.0 (3.9)^c,e^	4.919	0.002
HR, bpm	82.85 (15.94)	81.86 (15.22)	82.82 (15.35)	82.47 (16.05)	84.29 (17.10)	0.729	0.535
SBP, mmHg	121.81 (22.73)	120.07 (22.19)	121.04 (22.4)	122.71 (23.98)	123.55 (22.25)	0.835	0.475
DBP, mmHg	76.31 (14.34)	75.04 (13.91)	75.49 (13.69)	76.21 (15.64)	78.51 (13.91)	2.014	0.111
Medical history							
Diabetes duration, years	6.84 (1–10)	5.00 (1.75–10.00)	5.00 (2.00–10.00)	5.00 (2.00–10.00)	4.00 (1.00–10.00)	0.998	0.198
CKD	32 (4.60%)	7 (4.02%)	4 (2.31%)	9 (5.17%)	12 (6.90%)	1.472	0.220
Stroke	86 (12.37%)	28 (16.09%)	17 (9.83%)	20 (11.49%)	21 (12.07%)	1.130	0.335
Hypertension	445 (64.03%)	114 (65.52%)	108 (62.43%)	111 (63.79%)	112 (64.37%)	0.124	0.946
Dyslipidemia	295 (24.45%)	47 (27.01%)	58 (33.53%)^a^	83 (47.70%)^b,d^	107 (61.49%)^c,e,f^	8.245	<0.001
Smoking status						0.223	0.837
Former	70 (10.07%)	79 (45.40%)	83 (47.98%)	80 (45.98%)	78 (44.83%)		
Never	320 (46.04%)	22 (12.64%)	17 (9.83%)	17 (9.77%)	14 (8.05%)		
Now	305 (43.88%)	73 (41.95%)	73 (42.20%)	77 (44.25%)	82 (47.13%)		
Medication used before admission							
Antiplatelet drugs	80 (11.51%)	27 (15.52%)	13 (7.51%)	19 (10.92%)	21 (12.07%)	1.391	0.135
ACEI/ARB	130 (18.71%)	36 (20.69%)	31 (17.92%)	30 (17.24%)	33 (18.97%)	0.395	0.856
Beta-blocker	65 (9.35%)	18 (10.34%)	18 (10.40%)	16 (9.20%)	13 (7.47%)	0.467	0.763
Statins	22 (3.17%)	3 (1.72%)	3 (1.73%)	6 (3.45%)	10 (5.75%)	0.544	0.104
Hypoglycemic drugs							
Metformin	288 (41.44%)	64 (37.21%)	74 (42.77%)	69 (39.66%)	81 (46.55%)	0.329	0.322
SLG-2 inhibitor	9 (1.29%)	2 (1.16%)	3 (1.73%)	2 (1.15%)	2 (1.15%)	1.213	0.952
GLP-1 agonist	3 (0.43%)	0 (0.00%)	1 (0.58%)	1 (0.57%)	1 (0.57%)	1.438	0.803
Insulin	165 (23.74%)	33 (19.19%)	41 (23.70%)	52 (29.89%)	39 (22.41%)	0.631	0.124
Else	193 (27.77%)	38 (22.09%)	57 (32.95%)	45 (25.86%)	53(30.46%)	2.157	0.111

Values are presented as mean (SD), number (%), or median (interquartile range).

*P*-values stand for: ^a^Q2 compared to Q1 (*P* < 0.05); ^b^Q3 compared to Q1 (*P* < 0.05); ^c^Q4 compared to Q1 (*P* < 0.05); ^d^Q3 compared to Q2 (*P* < 0.05); ^e^Q4 compared to Q2 (*P* < 0.05); ^f^Q3 compared to Q4 (*P* < 0.05).

BMI, body mass index; HR, heart rate; SBP, systolic blood pressure; DBP, diastolic blood pressure; CKD, chronic kidney disease; MI, myocardial infarction; PCI, percutaneous coronary intervention; ACEI/ARB, angiotensin-converting enzyme inhibitor/angiotensin receptor blocker; SLG-2 inhibitor: sodium-dependent glucose transporter inhibitor; GLP-1 agonist: glucagon-like peptide-1 receptor agonist.

**Table 2:** Baseline levels of laboratory and angiographic characteristics according to the TyG index quartiles.

Characteristics	Quartiles of the TyG index				*F*-value	*P*-value
	Q1 (6.25–8.15)	Q2 (8.15–8.68)	Q3 (8.68–9.29)	Q4 (9.29–11.34)		
TyG index	7.66 (0.36)	8.43 (0.16)^a^	8.98 (0.17)^b,d^	9.79 (0.40)^c,e,^^f^	1,647.433	<0.001
SPISE index	8.31 (1.63)	7.42 (1.65)^a^	6.74 (1.52)^b,d^	5.64 (1.37)^c,e,f^	69.275	<0.001
HbA1c,%	7.35 (1.58)	7.90 (1.69)^a^	8.50 (1.84)^b,d^	8.83 (1.93)^c,e^	20.108	<0.001
eGFR, ml/min/1.73 m^2^	91.31 (35.54)	96.61 (41.41)	100.38 (42.77)	102.91 (41.50)	2.703	0.083
LDL cholesterol, mmol/L	2.13 (0.70)	2.23 (0.71)	2.28 (0.73)	2.11 (0.78)	1.545	0.202
HDL cholesterol, mmol/L	1.23 (0.64)	1.13 (0.66)	1.11 (0.63)	1.14 (0.65)	1.178	0.014
TC, mmol/L	3.96 (0.88)	4.06 (0.83)	4.20 (1.04)	4.58 (1.06)^c,e,f^	14.195	<0.001
TG, mmol/L	0.81 (0.31)	1.42 (0.52)^a^	2.05 (0.74)^b,d^	3.99 (2.37)^c,e,f^	202.449	<0.001
FPG, mmol/L	8.50 (2.83)	10.52 (3.73) ^a^	12.58 (4.43)^b,d^	15.34 (5.85) ^c,e,f^	78.507	<0.001
Albumin, g/L	37.40 (4.41)	38.25 (3.97)	38.18 (4.93)	39.70 (4.71) ^c,e,f^	7.771	<0.001
WBC, 10^9^/L	11.12 (3.28)	11.25 (3.89)	11.13 (3.90)	11.92 (4.28)	1.702	0.165
Hemoglobin, g/L	122.40 (50.93)	117.24 (53.76)	100.99 (62.50) ^b,d^	108.45 (63.98)	4.628	0.003
LVEF,%	49.80 (9.66)	50.40 (9.70)	50.34 (8.29)	50.03 (8.85)	0.156	0.926
Angiographic features						
Infarction related artery						
LM	0 (0.00%)	1 (0.58%)	1 (0.57%)	0 (0.00%)	0.668	0.570
LAD	88 (50.57%)	80 (46.24%)	88 (50.57%)	66 (37.93%)	2.499	0.059
RCA	71 (40.80%)	74 (42.77%)	68 (39.08%)	87 (50.00%)	1.636	0.179
LCX	15 (8.62%)	19 (10.98%)	18 (10.34%)	21 (12.07%)	0.384	0.763
No-reflow	5 (2.87%)	9 (5.20%)	13 (7.47%)	32 (18.39%) ^c,e,f^	10.994	<0.001
Thrombus aspiration	19 (10.92%)	20 (11.56%)	17 (9.77%)	27 (15.52%)	3.249	0.378
IABP	8 (4.60%)	15 (8.67%)	9 (5.17%)	15 (8.62%)	1.058	0.268
Bifurcation lesion	0 (0.00%)	0 (0.00%)	2 (1.15%)	1 (0.57%)	1.327	0.299
Calcifying lesion	1 (0.57%)	2 (1.16%)	2 (1.15%)	0 (0.00%)	2.078	0.527
Long lesion	15 (8.67%)	17 (9.83%)	18 (10.34%)	27 (15.52%)	1.623	0.182
Open lesion	10 (5.75%)	6 (3.47%)	14 (8.05%)	17 (9.77%)	2.078	0.102
Number of stents ≥2	54 (31.03%)	52 (30.06%)	49 (28.16%)	53 (30.46%)	0.489	0.751

Data are presented as mean (SD) or *n* (%).

*P-*values stand for:^a^Q2 compared to Q1 (*P* < 0.05); ^b^Q3 compared to Q1 (*P* < 0.05); ^c^Q4 compared to Q1 (*P* < 0.05); ^d^Q3 compared to Q2 (*P* < 0.05); ^e^Q4 compared to Q2 (*P* < 0.05); ^f^Q3 compared to Q4 (*P* < 0.05).

TyG, triglyceride–glucose index; TG, triglyceride; SPISE index, the single-point insulin sensitivity estimator; WBC, white blood cell; FPG, fasting plasma glucose; HbA1c, glycated hemoglobin; eGFR, estimated glomerular filtration rate; TC, total cholesterol; LVEF, left ventricular ejection fraction; LM, left main coronary artery; LAD, left anterior descending; LCX, left circumflex coronary artery; RCA, right coronary artery; IABP, intra-aortic balloon pump.

### Relationships between TyG index and no-reflow

Table 3 presents the occurrence of no-reflow in 59 patients during emergency PCI. We constructed multivariate regression models to investigate the independent association between the level of TyG index and no-reflow phenomenon. Our findings suggested that a higher TyG index was associated with increased no-reflow. The correlation between TyG index and no-reflow was significant in both our crude model (OR = 2.48, 95% CI: 1.76–3.50), *P *< 0.001) and the minimally adjusted model (OR = 2.96, 95% CI: 2.03–4.3, *P* < 0.001). The positive association between TyG index and no-reflow remained stable in the fully adjusted model (OR = 3.23, 95% CI: 2.15–4.86, *P *< 0.001), which indicated that each unit increase in TyG index was associated with a 2.23-fold increase in the risk of no-reflow.

**Table 3:** ORs (95% CIs) for no-reflow according to the TyG index quartiles.

No-reflow	*β* (95%CI) *P*-value Model 1	*β* (95%CI) *P*-value Model 2	*β* (95%CI) *P*-value Model 3
TyG index	2.48 (1.76, 3.50) <0.001	2.96 (2.03, 4.30) <0.001	3.23 (2.15, 4.86) <0.001
TyG index quartile			
Q1	Reference	Reference	Reference
Q2	1.85 (0.61, 5.65) 0.277	1.96 (0.64, 6.01) 0.238	2.29 (0.73, 7.15) 0.155
Q3	2.73 (0.95, 7.83) 0.062	3.03 (1.04, 8.77) 0.041	3.60 (1.21, 10.65) 0.021
Q4	7.62 (2.89, 20.06) <0.001	9.75 (3.63, 26.23) <0.001	12.28 (4.36, 34.62) <0.001

Model 1: non-adjusted.

Model 2: adjusted for age and gender.

Model 3: adjusted for age, gender, BMI, hypertension, dyslipidemia, CKD, smoking status, HDL cholesterol, TC, and albumin. OR, odds ratio; CI, confidence interval.

**Figure 2:**
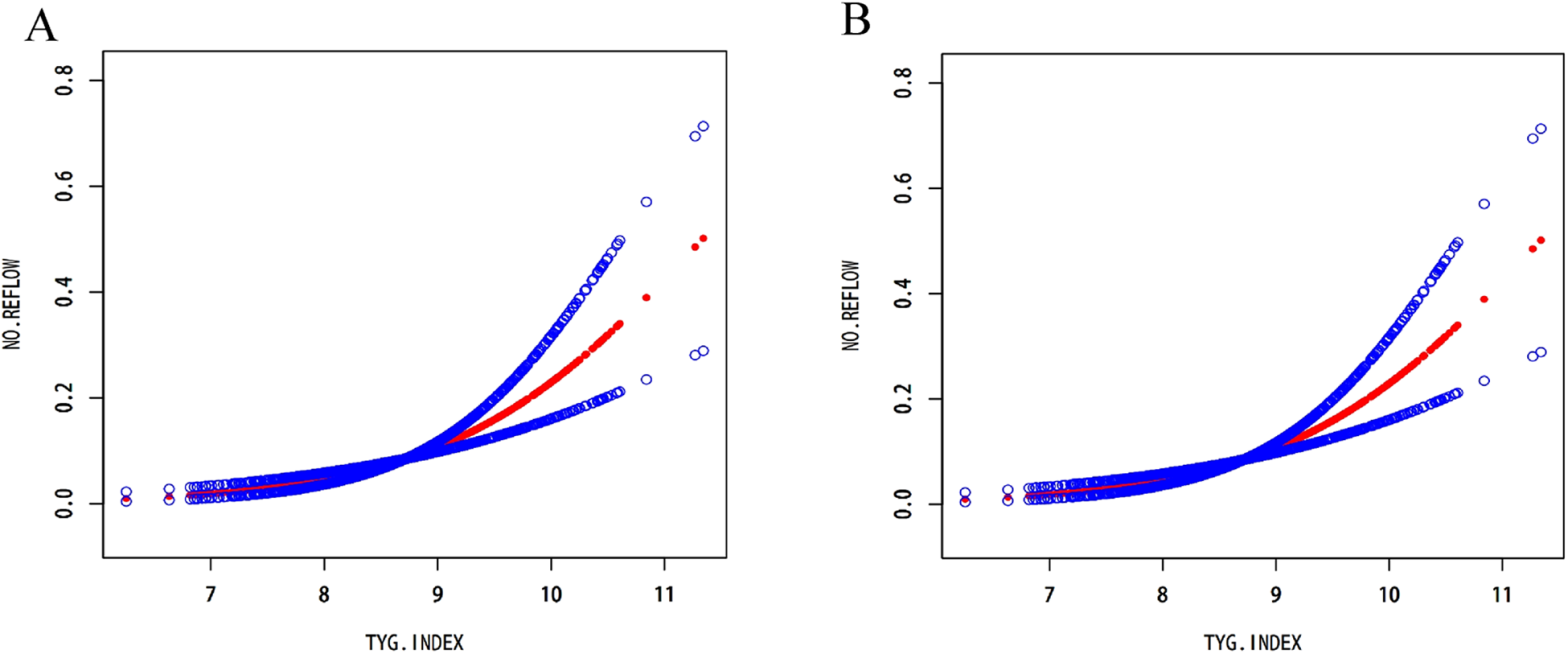
Analysis of the relationship between the TyG index and no-reflow by smooth curve fitting. The red line in the middle represented the fit line, and the line formed by the blue circles on both sides represented CI. **(A)** Smooth curve fitting analysis of the relationship between the TyG index and no-reflow did not correct any factors. **(B)** After adjusting for age, gender, hypertension, dyslipidemia, CKD, smoking status, BMI, HDL cholesterol, TC, and albumin, the relationship between the TyG index and no-reflow was analyzed by smooth curve fitting.

When the authors considered TyG index as tertiles, the participants in the highest tertile had an 11.28-fold increased risk of no-reflow compared to that of the participants in the first tertile (OR = 13.28, 95% CI: 4.44–39.71, *P* < 0.001). Additionally, the smooth curve fitting showed that TyG index is linearly related to the risk of no-reflow (Figure 2A). After adjusting for age, gender, hypertension, dyslipidemia, chronic kidney disease (CKD), smoking status, BMI, HDL cholesterol, TC, and albumin, the weighted smooth curve fitting also showed that TyG index was still linearly related to the risk of no-reflow (Figure 2B).

### Independent association of TyG index with no-reflow

**Figure 3:**
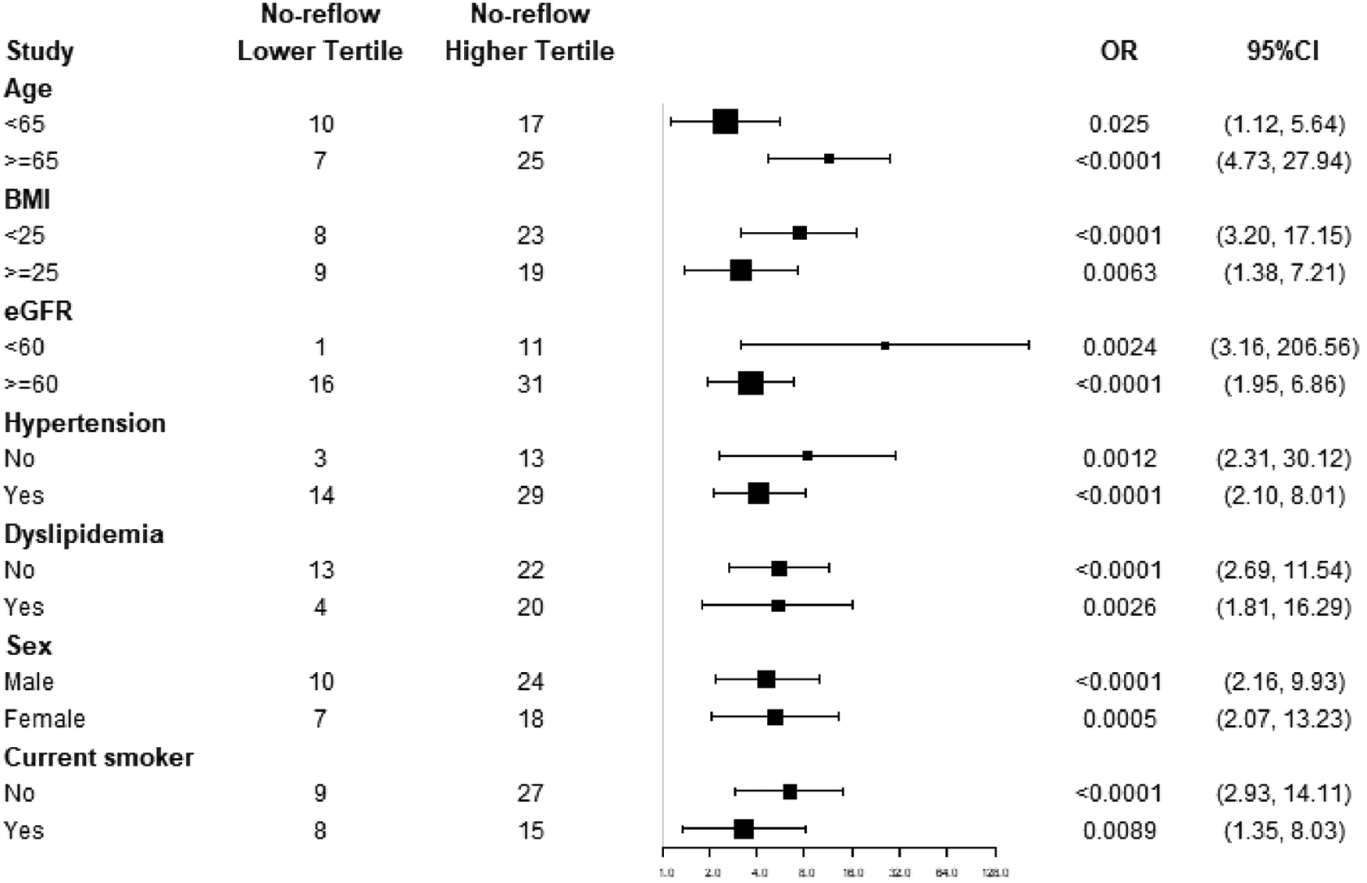
Forest plot of no-reflow according to different subgroups. The adjusted model included age, gender, hypertension, dyslipidemia, CKD, smoking status, BMI, HDL cholesterol, TC, and albumin.

A subgroup analysis was performed according to age, sex, BMI, current smoker, hypertension, dyslipidemia, and eGFR (Figure 3). We found that the predictive effect of TyG index on no-reflow is effective in most subgroups.

### Receiver operating characteristic (ROC) curve analyses to evaluate no-reflow

**Figure 4:**
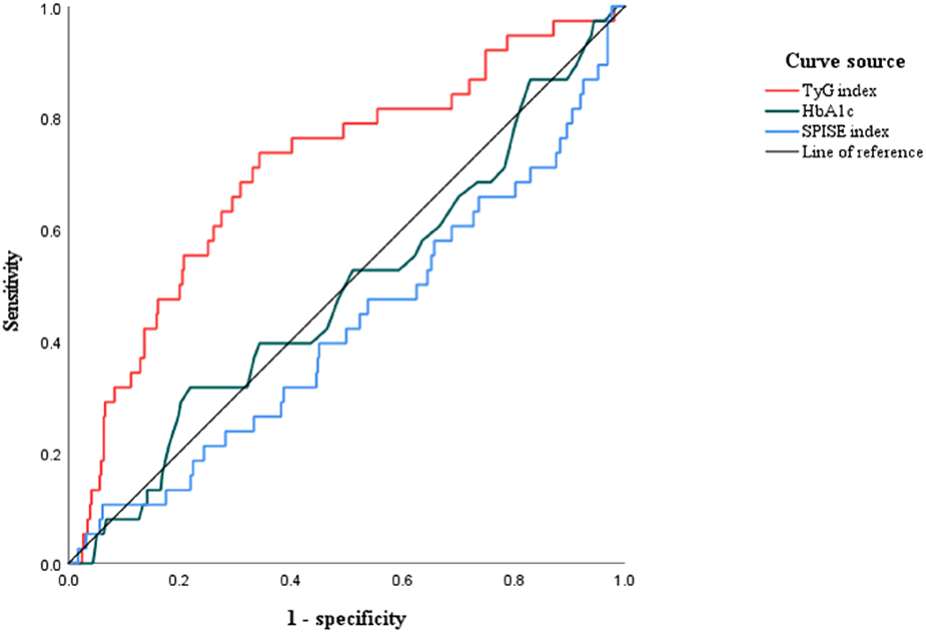
The receiver operating characteristic (ROC) curves of the TyG index, HbA1c, and the SPISE index to evaluate no-reflow in STEMI patients with T2DM. The area under the ROC curve (AUC) of the TyG index for evaluating the occurrence of no-reflow was 0.710 (95% CI: 0.633–0.771; *P < *0.01).

The area under the ROC curves (AUCs) of the TyG index for evaluating the occurrence of no-reflow was 0.710 (95% CI: 0.640–0.780; *P < *0.01). The cutoff value of the TyG index to evaluate no-reflow was 8.99, the sensitivity was 0.712, and the specificity was 0.665. The AUCs of HbA1c for evaluating the occurrence of no-reflow was 0.490 (95% CI: 0.392–0.589; *P *= 0.845). The cutoff value of the SPISE index to evaluate no-reflow was 0. 423 (95% CI: 0.323–0.524; *P *= 0.117) (Figure 4).

## Discussion

To the best of our knowledge, this is the first study to explore the association between the TyG index and no-reflow in STEMI patients with T2DM after PCI. Our main findings include the following: (1) The incidence of no-reflow increased significantly as the TyG index increased; (2) the TyG index was a univariate risk factor of no-reflow; (3) the association of the TyG index with no-reflow was mainly reflected in most subgroups; and (4) the AUC of the TyG index for evaluating the occurrence of no-reflow was 0.710 with a cutoff value of 8.99. Based on the present study, we found that the TyG index was linearly associated with no-reflow. Most importantly, this study suggests a simple method of estimating the association of IR and the risk of no-reflow after PCI in STEMI patients with T2DM.

IR is the decrease in the efficiency of insulin to stimulate the uptake and use of glucose, and the body responds by overproducing insulin, causing hyperinsulinemia, to maintain stable blood glucose levels. IR contributes to the development of cardiovascular diseases by inducing imbalances in glucose metabolism, generation of oxygen free radicals, reduction of nitric oxide production in endothelial cells, and alteration of systemic lipid metabolism, among other mechanisms (14). Several previous studies have found that IR is a significant risk factor for cardiovascular diseases and poor clinical outcomes (15, 16). At present, the complexity and cost of using the hyperinsulinemic–euglycemic clamp and HOMA-IR to detect IR make them inappropriate for widespread clinical use. To address this clinical challenge, researchers have identified the TyG index as a practical and effective alternative indicator of IR from numerous studies (17, 18). Therefore, the TyG index can be used in clinical practice to identify IR if the hyperinsulinemia–glucose clamp test and HOMA-IR are not measurable.

A large number of previous studies have provided strong evidence for the predictive role of the TyG index in diabetes and cardiovascular disease and for a poor prognosis. Zhao et al. (19) showed that an elevated TyG index is positively associated with a higher risk of atherosclerosis and renal microvascular damage. A study has also shown that the cumulative risk of developing T2DM increases with an increase in the TyG index (20). Mao et al. (21) first demonstrated that a higher TyG index was independently associated with SYNTAX score [OR (95% CI): 6.06 (2.92, 12.58), *P *< 0.001] and major adverse cardiovascular events [HR (95% CI): 1.79 (1.05, 3.07), *P *= 0.034] in the NSTE-ACS population. Additionally, a present study indicated that an increased TyG index is associated with an increased risk of major adverse cardiovascular events in STEMI patients undergoing PCI (11), and the risk of ischemic stroke is associated with a proportional linear increase in the TyG index (12). As almost one-third of patients with AMI have T2DM, these patients have more complex coronary disease, higher rates of recurrent adverse events, and a worse prognosis. To date, studies on the TyG index for the prediction of adverse cardiovascular events in patients with AMI in combination with T2DM have been published. A study by Ma and colleagues involving 776 patients with T2DM and ACS treated with PCI found that the TyG index was consistently associated with adverse cardiovascular outcomes, including total mortality, non-fatal stroke, non-fatal MI, and unplanned repeat revascularization (22). Furthermore, a study of 798 NSTE-ACS patients with T2DM showed a 2.208-fold increased risk of recurrent all-cause death, non-fatal MI, and ischemia-driven revascularization per one unit increase in TyG index [HR (95%CI): 3.208 (2.40–4.29), *P *< 0.001] and found that adding the TyG index to the baseline risk model had an incremental effect on the predictive value of poor cardiovascular prognosis (AUC: baseline risk model, 0.800 vs. baseline risk model + TyG index, 0.856, *P *< 0.001) (23). However, the association of the TyG index on no-reflow in STEMI patients with T2DM remains unclear.

In STEMI, acute hyperglycemia is a predictor of the occurrence of no-reflow and usually occurs as a result of a sudden increase in catecholamine levels. However, stress hyperglycemia may not be representative of a true acute glycemic state as it is also influenced by chronic blood glucose levels, particularly in diabetic patients (24). Recent studies (25) have shown that metrics combining acute and chronic blood glucose levels are more truly predictive of no-reflow than stress hyperglycemia. HbA1c is an important marker used in the diagnosis and treatment modalities of diabetes mellitus and is closely related to its complications and prognoses. HbA1c indicates an individual's blood sugar regulation in the last 3 months. Only a few studies have shown that HbA1c may be associated with no-reflow (26). Compared to stress hyperglycemia and HbA1c, the TyG index, consisting of lipid-related factors, glucose-related factors, and inflammation-related factors, may be a more reliable relevance of no-reflow in terms of the mechanism of no-reflow. In this study, we investigated for the first time the association of the TyG index with no-reflow after PCI in STEMI patients with T2DM. To gain a better understanding of the association of the TyG index with no-reflow, we analyzed and compared the correlation between different levels of the TyG index and no-reflow, which has not been attempted in other studies. In addition, we have also conducted the association of the TyG index with no-reflow in different subgroups, including age, gender, BMI, smoker, hypertension, eGFR, and dyslipidemia. We found that the TyG index has a favorable evaluating value for no-reflow in most subgroups.

Recent studies have shown that SGLT2 inhibitors (SGLT2i) reduce atherosclerotic plaque thickness, macrophage infiltration, and lipid arc, enhancing plaque stability in diabetic patients (27). SGLT2i also reduces intra-stent restenosis post-AMI, improving outcomes after PCI through glycemic control and anti-inflammatory effects, potentially lowering no-reflow incidence (28). Combining SGLT2i with GLP1 receptor agonists (GLP1-RA) enhances cardiac function, reduces hospitalizations, and improves survival in post-AMI patients (29). Given the link between no-reflow, inflammation, and vascular dysfunction, these drugs may reduce no-reflow. While our study did not include SGLT2i or GLP1-RA, their potential to improve no-reflow outcomes is acknowledged. Future research should explore their effects on myocardial perfusion and cardiovascular outcomes, underscoring the need for comprehensive management of T2DM patients undergoing PCI for STEMI.

There are limitations to our study. First, the generalization of the findings should be made with caution because this was a single-center study with a small sample size. Second, the laboratory parameters were only measured for the first time and lacked dynamic monitoring. This may introduce potential bias due to measurement error. Third, the TyG index for assessing the correlation with no-reflow after PCI in patients with STEMI combined with T2DM could not be directly compared with experimental methods such as HOMA-IR. Moreover, because our study did not have follow-up results, we could not draw conclusions about the prognostic relationship between TyG and clinical outcomes in STEMI patients with T2DM. Finally, as the TyG index is derived from fasting triglyceride and glucose levels, this parameter is not available for patients before PCI. Therefore, it can not predict the risk of no-reflow in non-fasting patients. Prospective cohort studies are needed to confirm our findings.

## Conclusions

Our results indicate that the TyG index is closely associated with the risk of no-reflow after PCI in patients with STEMI and T2DM and that the relationship between the TyG index and no-reflow incidence is linear. Therefore, the measurement of the TyG index may be useful in the assessment of risk and in the evaluation of prognosis in this patient population.

## Data Availability

The original contributions presented in the study are included in the article/Supplementary Material, further inquiries can be directed to the corresponding authors.
